# Camellia Oil Oleogels Structured with Walnut Protein–Chitosan Complexes: Preparation, Characterization, and Potential Applications

**DOI:** 10.3390/gels12010020

**Published:** 2025-12-25

**Authors:** Jun An, Liyou Zheng, Shuzhen Xuan, Xinyi He, Tao Yang

**Affiliations:** 1 College of Food Science and Engineering, Central South University of Forestry and Technology, Changsha 410004, China; anjunyang@126.com; 2 State Key Laboratory of Utilization of Woody Oil Resources, Central South University of Forestry and Technology, Changsha 410004, China; 3 College of Food Science and Engineering, Tianjin Agricultural University, Tianjin 300200, China; xsz0765@163.com (S.X.); hedevid@163.com (X.H.); 4 School of Biological and Food Engineering, Anhui Polytechnic University, Wuhu 241000, China; zhengliyou@ahpu.edu.cn

**Keywords:** cake, camellia oil, chitosan, walnut protein isolate, oleogel, stability, structural characteristics

## Abstract

As a step towards the substitution of saturated fats with camellia oil in foods, a camellia oil-based oleogel was prepared using a walnut protein isolate–chitosan (WPI-CS) composite via an emulsion template method. The preparation process, structural characteristics, and stability of the oleogel were systematically analyzed. Our findings showed that varying the ratio of WPI-CS to camellia oil (CO) effectively regulated the emulsion particle size, zeta potential, and viscosity, thereby subsequently influencing the oil-holding capacity (OHC), rheological properties, and thermal stability of the oleogel. When the WPI-CS:CO ratio was 13:7, the oleogel exhibited superior performance, including relatively high OHC, improved rheological properties, and excellent thermal stability. In addition, the OHC of the oleogel varied significantly with temperature, and high oxidative stability was observed at WPI-CS ratios such as 13:7–10:10. Application tests in cake formulations demonstrated that the oleogel has potential as a partial butter replacement. This study provides a theoretical basis for the construction of WPI-based oleogels and offers new insights for the development of healthy fat substitutes.

## 1. Introduction

Oleogels have attracted considerable attention due to their potential to convert liquid oils into solid fats while eliminating trans fats, reducing saturated fatty acid contents, and improving polyunsaturated fatty acid contents [1]. Camellia oil (CO)—an ideal base oil—is rich in bioactive compounds, including oleic acid, tocopherols, polyphenols, phytosterols, saponins, and squalene [2]. Meanwhile, CO, known as “oriental olive oil”, has gained popularity in Chinese cuisine. These bioactive compounds endow CO with a range of health benefits, such as antioxidant, anti-inflammatory, neuroprotective, hepatoprotective, and antimicrobial activities, along with the ability to mitigate liver damage, improve glycemic control, and exhibit anticancer properties [2,3]. Thus, CO has broad application prospects in the field of food, cosmetics and pharmaceuticals for the increasing population health needs [4]. However, prior to the use of CO in oleogels for replacing solid fats, CO must be integrated within a three-dimensional network.

In the development of a three-dimensional network, protein–polysaccharide interactions are particularly effective at stabilizing emulsions and preventing droplet coalescence, which is essential for forming high-internal-phase emulsions, emulsion gels, and, ultimately, oleogels [5,6,7,8]. Chitosan (CS), a naturally cationic polysaccharide with Generally Recognized as Safe (GRAS) status [9], is non-toxic, biocompatible, and biodegradable, with certain important biological properties such as antimicrobial properties [10]. CS becomes amphiphilic under acidic conditions, allowing it to adsorb at the oil–water interface and exhibit emulsifying properties, particularly when complexed with proteins. The increasing global demand for protein is driving the widespread use of plant protein as a valuable nutritional component, due to its excellent nutritional and health benefits. Walnut protein isolate (WPI) contains 18 types of amino acids, including a well-balanced profile of essential amino acids for the human body [11], and is an underutilized byproduct of walnut oil production [11,12]. The functionality of a protein is determined by a combination of its molecular properties (e.g., structure and hydrophobicity) and its interactive behavior with other constituents such as proteins and other components. Specifically, WPI and CS can interact through non-covalent forces (e.g., electrostatic attraction) and covalent bonding (via the Maillard reaction). Complexing WPI with CS combines their functional properties and provides an effective strategy to improve protein performance [9,13,14].

The aim of this complexation is to form oleogels—structured systems that convert liquid oils into solid fats without the use of trans fats or high levels of saturated fats [15,16,17,18]. This approach aligns with modern nutritional trends, offering healthier fat alternatives without compromising sensory quality [19,20,21]. Direct dispersion and emulsion templating are the two main methods for preparing oleogels [22]. It is reported that proteins with high surface activity are able to stabilize emulsions and further be used to fabricate oleogels via the emulsion templating approach [1]. In addition, the emulsion templating approach has shown great potential for food applications [8,16,23,24,25,26,27,28].

Currently, several oleogels using camellia oil as a base oil have been developed and applied in food products [22,29,30,31,32]. For instance, J. Li et al. (2023) [30] developed chitosan–gelatin cryogel templates, which were then immersed in a camellia oil bath to form oleogels. Jing et al. (2022) [32] also prepared a camellia oil-based oleogel with beeswax as the oleogelator, which was then applied in ice cream. However, studies on CS-stabilized WPI and its application in preparing WPI-CS composite CO (WCCO) oleogels are limited, and the research on camellia oil-based oleogel is still in its infancy. There is a particular lack of systematic investigations into the structure–activity relationship of WPI-CS complexes and their impact on the multiscale structural characteristics of WCCO oleogels.

Therefore, this study aimed to construct WPI-CS-stabilized CO emulsions (WCCEs) in order to prepare WCCO oleogels via the emulsion templating approach. The emulsions, intermediate freeze-dried samples, and final oleogels were characterized in terms of their texture, morphology, oil-holding capacity, and stability. Furthermore, the WCCO oleogels were tested as partial butter substitutes in cake formulations, and the sensory and textural properties of the cakes were evaluated. This work provides theoretical and empirical foundations for developing WPI-based oleogels with food applications.

## 2. Results and Discussion

### 2.1. Analysis of WCCEs

#### 2.1.1. Mean Particle Size, Zeta Potential, and Viscosity

**Figure 1:**
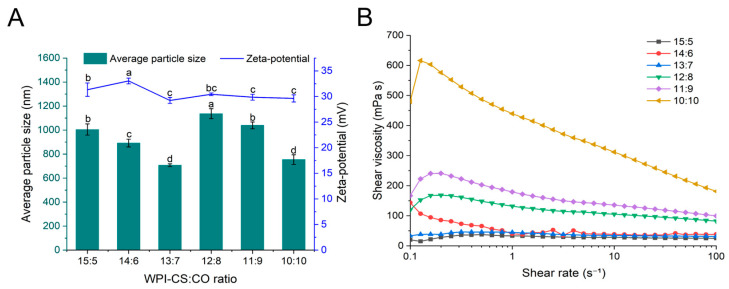
Mean grain size and zeta potential (**A**), and viscosity (**B**) of OBEs. Different letters indicate significant differences among samples (*p* < 0.05).

Emulsion samples were prepared using WPI-CS and CO at ratios of 15:5, 14:6, 13:7, 12:8, 11:9, and 10:10. WCCO oleogels were then produced using the emulsion templating method. The zeta potential and particle size are key parameters, not only for characterizing emulsions but also for determining the stability of oleogels prepared via the emulsion templating method [24]. The particle size and zeta potential of the WCCEs were first analyzed and are shown in Figure 1A. As the amount of CO increased, the mean particle size of the emulsions initially decreased significantly, then increased, and finally decreased again. This fluctuation could be attributed to the effects of the CO concentration on emulsifier coverage [33]. At low CO concentrations, WPI-CS could fully cover the surface of the CO droplets, forming smaller droplets. However, at high CO concentrations (13:7 → 12:8), the emulsifier was insufficient to completely cover CO droplets, resulting in droplet aggregation and increased particle size. When the WPI-CS:CO ratio was 12:8, the mean particle size of the WCCEs reached a maximum of 1137 ± 41 nm, with a zeta potential of 30.43 ± 0.25 mV. In contrast, at a ratio of 13:7, the mean particle size of the emulsions was minimal at 707 ± 11 nm, with a zeta potential of 29.23 ± 0.59 mV. The zeta potentials of all emulsion samples were approximately 30 mV, indicating good emulsion stability. Notably, the zeta potentials exceeded 30 mV at WPI-CS:CO ratios of 15:5 and 14:6, suggesting excellent stability in these systems. According to DLVO theory, when the absolute value of the zeta potential exceeds 30 mV, electrostatic repulsion sufficiently overcomes van der Waals attraction, thereby preventing droplet aggregation [34]. At an aqueous-phase pH of 4, the WPI-CS complex was positively charged since WPI has an isoelectric point near pH 5 and the amino groups of CS are protonated under acidic conditions, enabling electrostatic adsorption on the CO surface. This formed a bilayer emulsion that exhibited both electrostatic repulsion and steric hindrance, thus preventing droplet aggregation [6,8].

The effects of the WPI-CS:CO ratio on the structural properties of WCCEs were investigated through viscosity measurements. For emulsion samples with WPI-CS:CO ratios of 10:10, 11:9, 12:8, and 14:6, their shear viscosity decreased significantly with increasing shear rate (*p* < 0.05), indicating pronounced shear-thinning behavior typical of pseudoplastic liquids [23]. This typical shear-thinning behavior could be attributed to shear-induced molecular rearrangement and disruption of the emulsion network [8,25]. Additionally, WCCEs prepared with WPI-CS:CO ratios of 10:10, 11:9, and 12:8 exhibited higher viscosities, possibly due to the formation of a network between WPI and CS, facilitated by electrostatic interactions between droplets. This network acted as a stabilizer in the bilayer emulsion [8]. The higher shear viscosities observed in these WCCEs also corresponded to greater mechanical disruption during homogenization, promoting the formation of smaller oil droplets [35].

#### 2.1.2. Morphology of WCCEs

**Figure 2:**
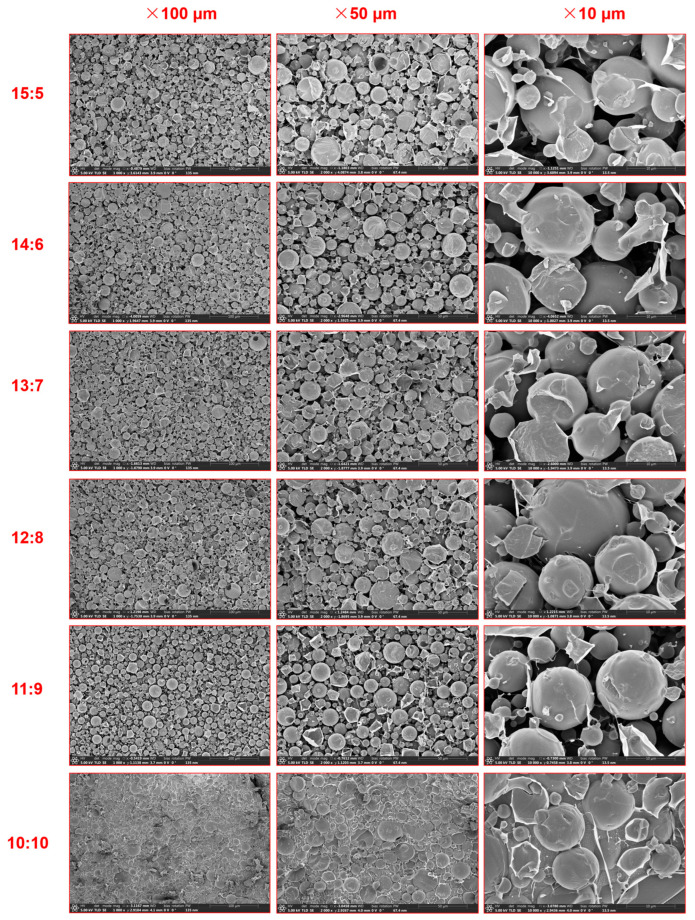
Cryo-SEM images of WCCEs.

Cryo-SEM images of the WCCEs are shown in Figure 2. At WPI-CS:CO ratios between 15:5 and 13:7, the emulsions exhibited smaller droplets (ranging from nanometers to micrometers in diameter) with a uniform and dense distribution. This indicated that the WPI-CS complex effectively reduced oil–water interfacial tension through electrostatic interactions and steric hindrance, thereby preventing the coalescence of oil droplets and forming a stable oil-in-water (O/W) emulsion. As the oil-phase proportion increased (especially at 11:9 and 10:10), there was a possibility for the interfacial film to rupture or become thinner, leading to droplet deformation. At WPI-CS:CO ratios of 11:9 and 10:10, droplet clustering or flocculation was observed, indicating that the electrostatic shielding or steric hindrance was insufficient, resulting in moderate emulsion instability [36]. All WCCEs samples had droplet particle sizes of around 1000 nm and zeta potential values of around 30 mv, which indicated that the WCCEs were suitable for the preparation of oleogels.

### 2.2. Analysis of Freeze-Dried Samples

#### 2.2.1. Visual Appearance of Freeze-Dried Samples

**Figure 3:**
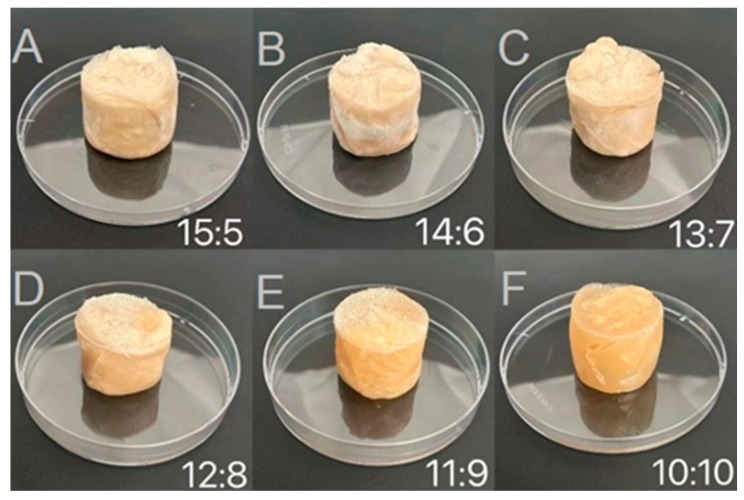
Visual appearance of freeze-dried samples. (**A**–**F**) denote samples with WPI-CS:CO ratios of 15:5, 14:6, 13:7, 12:8, 11:9, and 10:10, respectively.

Characterizing the performance of freeze-dried samples is of great significance because they are a key intermediate in the preparation of WCCO oleogels via the emulsion templating method. In this study, these samples were systematically analyzed. They were placed evenly in clean culture dishes and observed at a constant temperature of 20 °C (Figure 3). After 48 h of freeze-drying, samples with WPI-CS:CO ratios of 15:5 and 14:6 (Figure 3A,B) appeared as solid white cylinders with no visible surface oil [23]. When the WPI-CS:CO ratio exceeded 11:9, the samples demonstrated a softer texture, although surface oil remained minimal (Figure 3C,D). In contrast, for samples with WPI-CS:CO ratios of 11:9 and 10:10, ice crystals formed from water during the freeze-drying process and disrupted the WCCO structure [8], resulting in obvious oil leakage on the surface of these samples (Figure 3E,F). Similar results were also observed in soybean oil bodies–xanthan gum composite oleogels [8]. However, the WPI-CS complex continued to protect CO droplets from coalescence and reduced interfacial damage from ice crystals during freeze-drying, thereby maintaining the integrity of the WCCO structure. Samples prepared with WPI-CS:CO ratios of 13:7–11:9 seemed to have a better visual appearance compared with the other samples.

#### 2.2.2. TPA of Freeze-Dried Samples

**Figure 4:**
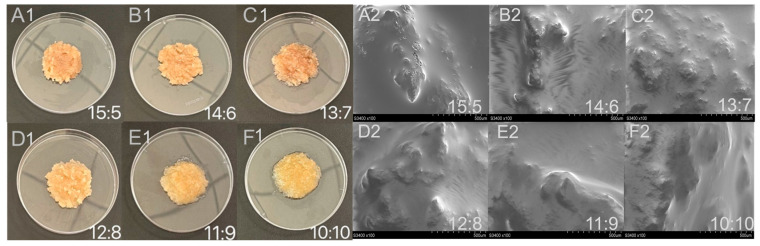
Visual appearance (**A1**–**F1**) and SEM images (**A2**–**F2**) of oleogels.

TPA was performed to evaluate the influence of varying the WPI-CS:CO ratio on the gel structure of freeze-dried samples, as depicted in Table 1. Hardness reflects the density of the three-dimensional network and indicates the gel strength of the WCCO oleogels. Elasticity and resilience represent the ability of the freeze-dried samples to return to their original structure after the removal of an external compressive force. As shown in Table 1, the hardness of the freeze-dried samples increased significantly (*p* < 0.05) with increasing WPI-CS:CO ratio, suggesting the formation of a more compact network in samples with higher WPI-CS:CO ratios [37]. These results agree with the visual appearance of the oleogels in Figure 4. However, the WPI-CS:CO ratio had no significant effect on the cohesiveness, elasticity, or resilience of the freeze-dried samples (*p* > 0.05).

### 2.3. Analysis of WCCO Oleogels

#### 2.3.1. Morphology of WCCO

Figure 4 presents the macroscopic morphology and microstructural characteristics of the WCCO oleogels prepared in this study. The oleogels, formed by shearing the freeze-dried samples, exhibited typical semi-solid characteristics and appeared as irregular particle aggregates with rough surfaces (Figure 4A1–E1). In contrast, samples with a WPI-CS:CO ratio of 10:10 exhibited a more liquid appearance and lower transparency, suggesting their denser structure (Figure 4F1). This was confirmed via SEM imaging, which showed a relatively smooth surface (Figure 4F2). However, as the WPI-CS:CO ratio increased from 11:9 to 15:5, the SEM images revealed an increasingly rougher oleogel surface, potentially due to the formation of a cross-linked network by the WPI-CS complex. For example, Figure 4A2 shows visible gaps on the oleogel surface. CO droplets were embedded in the three-dimensional network formed by the WPI-CS complex, consistent with the oleogel morphology described previously [8]. Above all, the samples prepared with WPI-CS:CO ratios of 13:7–11:9 seemed to have better visual appearances compared with the other samples.

#### 2.3.2. OHC of WCCO Oleogels

**Figure 5:**
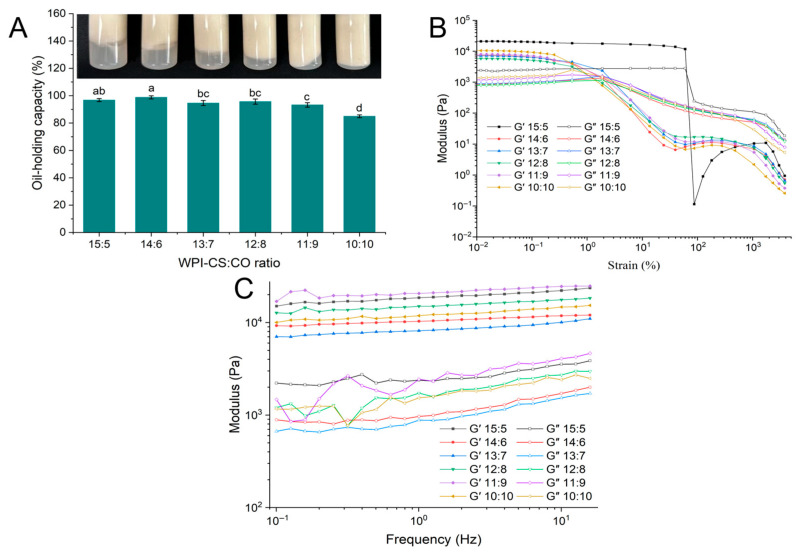
OHC (**A**) and rheological properties (**B**,**C**) of oleogels. Panels (**B**,**C**) represent amplitude sweep and frequency sweep, respectively. Different letters indicate significant differences among samples (*p* < 0.05).

OHC, which represents the ability of a structured network to retain oil, is a key parameter for evaluating the macroscopic properties of oleogels. Figure 5A illustrates the OHC of WCCO oleogels prepared with different WPI-CS:CO ratios. At a WPI-CS:CO ratio of 15:5, the OHC of the WCCO oleogel reached 96.79%, while at a 10:10 ratio, it decreased to 84.94%. The OHC of the oleogel exhibited a significant decreasing trend as the WPI-CS:CO ratio decreased (*p* < 0.05). This decline in OHC was attributed to the less dense and less stable three-dimensional network formed at WPI-CS:CO ratios such as 10:10. In contrast, at higher ratios (15:5–11:9), CO droplets were tightly encapsulated in the polymer matrix, resulting in enhanced mechanical stability and rupture resistance during freeze-drying and shearing processes [15,38].

#### 2.3.3. Rheological Properties of WCCO Oleogels

The rheological behavior of the WCCO oleogels was evaluated by measuring their storage modulus (G′) and loss modulus (G″) under strain and frequency sweeps, as shown in Figure 5B,C. In the shear strain range of 0.01% to 1%, G′ was consistently greater than G″ (Figure 5B), indicating that the oleogels exhibited viscoelastic solid behavior with higher gel strength in the linear viscoelastic region. This suggested that the elastic response dominated when the oleogels underwent deformation or external stress [1,38,39]. However, at higher shear strains (>1%), a distinct crossover point (G″ = G′) was observed, suggesting the beginning of structural breakdown in the oleogels under higher stresses [15]. When G″ exceeded G′, the structure of the oleogels was considered to be disrupted [1]. Frequency sweeps were conducted to assess the structural integrity and support properties of the oleogels. Figure 5C displays the frequency-dependent behavior of the G′ and G″ values of the oleogels. Across the frequency range of 0.1–10 Hz, all oleogels exhibited G′ values greater than their G″ values, with a weak frequency dependence of G′. A similar behavior has been reported in other emulsion-templated oleogels [23,24,25,40].

#### 2.3.4. Thermal Stability of WCCO Oleogels

**Figure 6:**
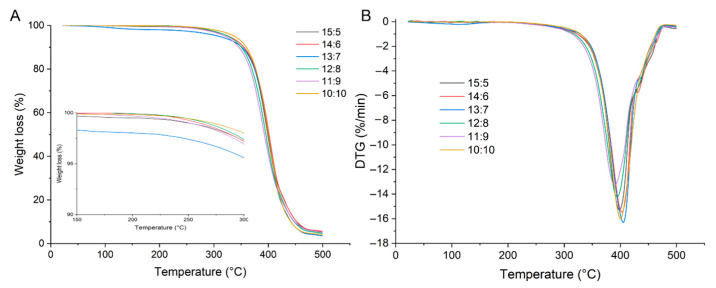
Thermal stability ((**A**), TGA; (**B**), DTG) of oleogels. Different letters indicate significant differences among samples (*p* < 0.05).

Figure 6A,B show the thermogravimetric analysis (TGA) and derivative thermogravimetric (DTG) curves of the WCCO oleogels. In general, the mass loss from the oleogels occurred in three degradation stages [39,41]. The first stage (25–113 °C) corresponded to the loss of water and low-molecular-weight volatiles. During this stage, all samples exhibited approximately 1% weight loss (Figure 6A), indicating that most of the water was effectively removed during freeze-drying. The second stage (113–480 °C) exhibited the most significant mass loss, primarily due to the depolymerization of the WPI-CS network and the carbonization of CO. The third stage (480–500 °C) involved the carbonization of degradation products [42] or thermal hysteresis of the oleogel structure [41]. The DTG curves of the oleogels (Figure 6B) revealed that the peak decomposition temperatures for WCCO oleogels with WPI-CS:CO ratios ranging from 15:5 to 10:10 were approximately 398, 402, 405, 394, and 400 °C, respectively. Comparisons among the TGA and DTG curves of these oleogels showed no significant differences in their residual mass or thermal decomposition rates.

### 2.4. Microstructure of Cake Batters

**Figure 7:**
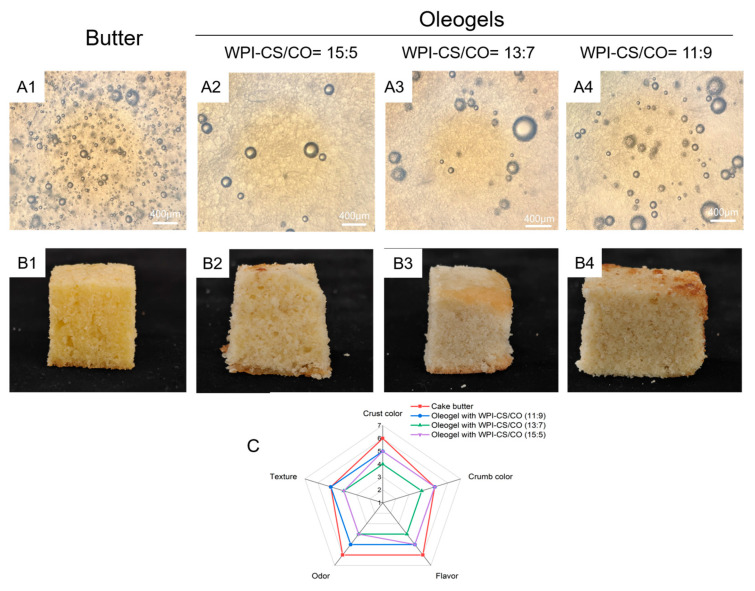
Light microscopy images of cake batters (**A1**–**A4**) and cakes prepared using oleogel emulsions and standard cake margarine as shortenings (**B1**–**B4**). Comparative sensory parameters of cakes prepared using oleogels and butter (**C**).

The difference in the microstructure of cake batters is shown in Figure 7. The number of bubbles in batters prepared with WCCO oleogels was lower than that in batters made with butter (Figure 7A1–A4), which could be attributed to the absence of fat crystals and emulsifiers in the oleogels [43,44]. Similar findings were reported in a prior study [44], who prepared cake batters using margarine, shortening, and oleogel. As the WIP-CS:CO ratio increased, the number of bubbles in the batter significantly decreased. This phenomenon could be explained by the increased mechanical strength of the batter due to the higher WIP-CS concentration in the WCCO oleogel, which prevented air incorporation during mixing [15].

### 2.5. Textural Analysis and Sensory Evaluation of Cakes

To evaluate the potential application of WCCO oleogels in bakery products, cakes were prepared using WCCO oleogels or butter (Figure 7B1–B4), and then TPA (Table 2) and sensory evaluations (Figure 7C) were conducted.

The internal structure of cakes made with WCCO oleogels was coarser than that of butter-based cakes (Figure 7B1–B4), and this coarseness increased with higher WPI-CS:CO ratios. Figure 7C presents sensory evaluation data on the crust color, crumb color, odor, flavor, texture, and overall quality. Compared to the control butter-based cakes, WCCO-based cakes demonstrated lower scores across all evaluation criteria, with especially low scores at WPI-CS:CO ratios of 13:7 and 11:9. The reduction in texture scores was particularly significant (*p* < 0.05). The overall quality score of the butter-based cakes was 32.03 points, while those of the WCCO-based cakes ranged from 21 to 27 points.

As shown in Table 2, cakes prepared with WCCO oleogels demonstrated harder textures and higher cohesiveness compared to the butter-based cakes. This might lie in the reason that the small amount of air bubbles in batters with oleogels would make it difficult for the cake to have a soft structure [18]. No significant differences were observed in their elasticity (*p* > 0.05), while their chewiness and stickiness were significantly increased (*p* < 0.05). Among the different WPI-CS:CO ratios, ratios of 13:7 and 11:9 resulted in lower cake resilience; this is possibly due to the formation of a gel network by the WCCO oleogel, which created new voids in the batter, thereby decreasing cake elasticity. There was a significant negative correlation between cake hardness and overall quality [44].

As the WPI-CS/CO ratio increased, cake hardness rose, contributing to lower sensory scores observed in WCCO-based cakes. Notably, there were no significant differences in odor scores across all cakes. In terms of overall acceptability, cakes prepared with lower WPI-CS:CO ratios (13:7 and 11:9) were considered acceptable by the sensory panel. Although complete replacement of butter with WCCO oleogels can significantly reduce the saturated fat content of baked products, producing food products without using solid fats presents challenges, as these fats are responsible for providing the desired texture, structure, and mouthfeel. Based on the texture and sensory evaluation results, producing high-quality cakes through the complete replacement of butter with WCCO oleogels appears challenging. Therefore, partial replacement of butter with these oleogels should be considered as an alternative approach to reduce trans and saturated fat contents while maintaining desirable physical and sensory properties in baked products.

## 3. Conclusions

This study examined the effects of various WPI-CS:CO ratios on the preparation and structural properties of WCCO oleogels. The WPI-CS complex effectively stabilized the CO emulsion. The three-dimensional network formed by WPI-CS encapsulated CO and significantly enhanced the OHC of the resulting WCCO oleogel. This oleogel exhibited high viscoelasticity and gel strength, contributing to a reduced oxidation rate of the oil. In addition, cakes prepared with WCCO oleogels had a similar color to those made with butter. While the aroma and taste of the WCCO-based cakes were slightly less favorable, their texture was comparable to that of the butter-based cakes. Future studies should further explore and optimize the oleogel preparation process to enable targeted regulation of physical and functional properties. Expanding the application of WCOO oleogels in the health-oriented food sector and evaluating their effects on the physical and sensory properties of food products are also recommended. Moreover, the application of WCCO in baked goods can be extended by incorporating supplementary ingredients to enhance flavor and enable complete butter replacement, offering advantages of lower saturated fat contents, reduced trans fatty acid contents, and increased protein levels, thereby improving the nutritional value of the product.

## 4. Materials and Methods

### 4.1. Materials

Walnut protein powder was obtained from Hebei Lehuo Vegetable Oil Co., Ltd. (Xingtai, China). Chitosan (Mn = 150–300 kDa, low viscosity: 100–200 mPa.s, degree of deacetylation ≥ 95%) was purchased from Aladdin, and CO was supplied by Anhui Huayin Tea Oil Co., Ltd. (Luan, China). Distilled water was used throughout all experimental procedures. Other chemicals, including glacial acetic acid, 2-propanol, and isooctane, were of analytical grade.

### 4.2. Extraction of WPI

Approximately 10 g of walnut protein powder was weighed. According to Ma et al. (2024) [11], this powder contains 9.93 ± 0.06% moisture, 77.60 ± 5.49% protein (dry basis, as quantified via the BCA method), and 2.12 ± 0.40% oil (dry basis). The powder was added to 150 mL of 50 °C ultrapure water, stirred using a magnetic stirrer, and adjusted to pH 9. The mixture was continuously stirred at 40–50 °C for 90 min. Subsequently, the supernatant was collected via centrifugation (4000 rpm, 20 min), and residual solids and impurities were removed through filtration with filter paper. It was then acidified to pH 4.6 using 50% glacial acetic acid and stirred with a magnetic stirrer for 60 min. The mixture was centrifuged (4000 rpm, 20 min) to collect the precipitate, which was then resuspended, magnetically stirred for 60 min, adjusted to pH 7, and diluted to 500 mL with deionized water to obtain a WPI solution with a concentration of approximately 10 mg/mL. This mother liquor was refrigerated for subsequent use.

### 4.3. Preparation of WCCEs, Freeze-Dried Samples, and Oleogels

Following the method described by Miao et al. (2025) [23], a 0.5% (*v*/*v*) acetic acid solution was prepared by adding 0.5 mL of glacial acetic acid to a 100 mL volumetric flask and making up the volume using distilled water. Subsequently, 1 g of CS powder was precisely weighed and added to 100 mL of the acetic acid solution, and the mixture was stirred continuously for 2–3 h at room temperature using a magnetic stirrer to obtain a 10 mg/mL CS stock solution, which was stored under refrigeration.

The WPI and CS solutions were mixed in a 1:1 mass ratio to prepare the aqueous phase of the emulsion. The solution was magnetically stirred at a constant temperature until thoroughly mixed, and its pH was adjusted to 4. CO was used as the oil phase. Emulsions were prepared using six different ratios of the aqueous phase to the oil phase: 15:5, 14:6, 13:7, 12:8, 11:9, and 10:10. The aqueous and oil phases were combined and homogenized at 14,000 rpm for 3 min using a disperser (IKA T 25 digital ULTRA-TURRAX, IKA-WERK, Staufen, Germany) to obtain WCCEs. These emulsions were frozen at −80 °C for 4 h and subsequently freeze-dried for 48 h using a vacuum freeze-dryer. The six resulting freeze-dried samples were sheared at 2000 rpm for 3 min using a Vorwerk Thermomix TM31 device (Vorwerk, Wuppertal, Germany) to produce the final oleogel products.

### 4.4. Characterization of WCCEs

#### 4.4.1. Mean Particle Size and Zeta Potential Measurements

The mean particle size and zeta potential of the WCEEs were measured using a Zetasizer Nano ZS (Malvern Instruments, Worcestershire, UK) at 25 °C. The refractive indices of the dispersed and continuous phases were set to 1.456 and 1.333, respectively.

#### 4.4.2. Viscosity Measurement

The viscosity of the WCCEs was measured using a HAAKE MARS 60 rheometer (Thermo Fisher Scientific, Waltham, MA, USA). Viscosity measurements were taken at shear rates ranging from 0.1 to 100 s^−1^. All the measurements were carried out at 25 °C.

#### 4.4.3. Morphological Observation

The morphology of the WCCEs was observed using cryo-scanning electron microscopy (cryo-SEM; SU8010, Hitachi, Tokyo, Japan) at an accelerating voltage of 5 kV, following the method reported by Tavernier et al. (2017) [45].

### 4.5. Characterization of Freeze-Dried Samples

#### 4.5.1. Visual Appearance Analysis

Freeze-dried samples were placed on Petri dishes and photographed against a black background in a photo box (Daehan Co., Incheon, Republic of Korea) equipped with an LED light source.

#### 4.5.2. Textural Analysis

Texture profile analysis (TPA) of freeze-dried samples was conducted using a TA.XT Plus C texture analyzer (Stable Micro Systems, Surrey, UK), following the procedures described by Z. Meng et al. (2018) [37] and Luo et al. (2019) [15], with some modifications. A cylindrical probe (P/5, diameter = 5 mm) was used to conduct compression-based TPA measurements at 20 °C. The pre-test, test, and post-test speeds were set to 5, 1, and 5 mm/s, respectively, with a trigger force of 5 g.

### 4.6. Characterization of Oleogels

#### 4.6.1. Morphological Observation

The oleogel morphology was observed using scanning electron microscopy (SEM; S3400, Hitachi, Tokyo, Japan) under vacuum at ambient temperature. The accelerating voltage was 5 kV [8].

#### 4.6.2. OHC Measurement

Following the method described by Miao et al. (2025) [23], 2 g of oleogel was centrifuged at 10,000 rpm for 15 min at a temperature below 20 °C. After inversion for 30 min, free oil was removed using filter paper. Each sample was tested in triplicate. The initial mass of the oleogel was designated M1, and the mass after free oil removal was denoted by M2. OHC was calculated using the following equation:

$$OHC\left(\%\right)=\frac{M_{2}}{M_{1}}\times 100$$

#### 4.6.3. Rheological Property Measurement

The rheological properties of the oleogels were analyzed using a HAAKE MARS 60 rheometer (Thermo Fisher Scientific, Waltham, MA, USA) equipped with parallel plates of 25 mm diameter and with a 2 mm gap. The linear viscoelastic region (LVR) and yield stress were determined by conducting amplitude and frequency sweeps, as described by Tao et al. (2025) [38]. The amplitude sweeps were performed from 0.01% to 1000% at 1 Hz and 20 °C. Meanwhile, the frequency sweeps (0.1–100 rad/s) were conducted within the LVR at 0.1% constant strain.

#### 4.6.4. Thermal Stability Measurement

The thermal stability of the oleogels was analyzed using an STA 2500 thermogravimetric analyzer (Netzsch, Selb, Germany) [39]. Each sample (5 mg) was heated from 25 to 500 °C at a rate of 10 °C/min.

### 4.7. Cake Preparation and Characterization

#### 4.7.1. Cake Preparation

Cakes were prepared according to methods described previously [15,43], with modifications to analyze the application of WCCO. Butter (Tianjin Nanqiao Food Co., Ltd., Tianjin, China) served as the control. WCCO oleogels prepared with WPI-CS:CO ratios of 15:5, 13:7, and 11:9 (Section 2.3) were used. The cake formulation included 100 g wheat flour, 100 g powdered sugar, 100 g liquid whole egg, 100 g butter or oleogel, and 1.3 g baking powder. The batter was baked at 175 °C for 25 min and then cooled at room temperature for 30 min before analysis.

#### 4.7.2. Microstructure of Cake Batter

The cake batter microstructure was observed using an XSP-2C optical microscope (Shanghai Optical Instrument Factory No. 6, Shanghai, China) at 160× magnification.

#### 4.7.3. TPA of Cakes

Cross-sectional images of the cakes were captured using a camera. TPA was performed using a texture analyzer (Stable MicroSystems Co., Ltd., Surrey, UK) according to the methods described by Luo et al. (2019) [18] and Patel et al. (2014a) [44]. The cake samples were cut into 3 cm × 3 cm × 3 cm cubes and compressed twice using a P/75 probe to 50% deformation. The test parameters included a trigger force of 5 g, a pre-test speed of 2 mm/s, a test speed of 1 mm/s, and a post-test speed of 5 mm/s. Seven measurements were performed on each cake, and the mean value was calculated.

#### 4.7.4. Sensory Evaluation of Cakes

Sensory evaluation was conducted using a 7-point scale, following the methods described by Luo et al. (2019) [18], with some modifications. Evaluators were recruited from a list of volunteers who regularly consumed and enjoyed cakes. Thirty untrained evaluators (thirteen males and seventeen females, aged 20–53 years) participated as informed volunteers and were briefed on how to assess various cake properties before testing. The evaluations were conducted in individual booths at room temperature. Evaluators assessed the cakes based on their crust color, aroma, taste, texture, crumb color, and overall acceptability. Each cake cube (3 cm × 3 cm × 3 cm) was placed on a plate labeled with a random three-digit code. Evaluators rinsed their mouths between samples to minimize carryover effects. Ratings were recorded using a 7-point scale: 1 = greatly dislike, 2 = very much dislike, 3 = somewhat dislike, 4 = neither like nor dislike, 5 = somewhat like, 6 = very much like, and 7 = greatly like.

### 4.8. Statistical Analysis

All tests were performed in triplicate, and the experimental results are expressed as the mean ± standard deviation (SD). Statistical analysis was conducted using SPSS 19.0 (SPSS Inc., New York, NY, USA). One-way analysis of variance (ANOVA) was used to assess the data, and Duncan’s multiple comparison test was employed to determine significant differences between mean values. Differences were considered statistically significant when *p* ˂ 0.05. Figures were generated using the OriginPro 8.0 software.

## Figures and Tables

**Table 1:** Texture profile analysis of freeze-dried samples.

WPI-CS:CO	Hardness (g)	Springiness (mm)	Cohesiveness (−)	Gumminess (g)	Chewiness (mJ)	Resilience (−)
15:5	170.19 ± 81.55 ^a^	0.44 ± 0.01 ^a^	0.41 ± 0.02 ^abc^	69.81 ± 8.02 ^c^	30.52 ± 5.01 ^c^	0.14 ± 0.02 ^ab^
14:6	182.9 ± 29.81 ^a^	0.41 ± 0.03 ^a^	0.44 ± 0.03 ^abc^	87.55 ± 1.31 ^b^	35.90 ± 0.09 ^c^	0.14 ± 0.02 ^ab^
13:7	172.9 ± 28.78 ^a^	0.42 ± 0.02 ^a^	0.37 ± 0.01 ^c^	104.00 ± 2.02 ^a^	43.08 ± 1.93 ^b^	0.13 ± 0.01 ^b^
12:8	94.01 ± 24.57 ^b^	0.45 ± 0.07 ^a^	0.40 ± 0.02 ^bc^	111.30 ± 5.89 ^a^	48.79 ± 0.08 ^a^	0.12 ± 0.01 ^b^
11:9	49.48 ± 20.04 ^b^	0.50 ± 0.02 ^a^	0.41 ± 0.03 ^abc^	81.69 ± 10.56 ^bc^	38.79 ± 7.49 ^bc^	0.16 ± 0.01 ^a^
10:10	36.24 ± 14.1 ^b^	0.49 ± 0.05 ^a^	0.46 ± 0.04 ^a^	78.13 ± 2.53 ^c^	42.84 ± 2.15 ^b^	0.14 ± 0.01 ^ab^

Note: Values denote means ± standard deviations of at least three determinations. Mean values in same column with different superscripts are significantly different (*p* < 0.05).

**Table 2:** Texture profile analysis of cake samples.

Sample	Hardness (g)	Springiness (mm)	Adhesiveness (mJ)	Cohesiveness (−)	Chewiness (mJ)	Resilience (−)
Butter	458.36 ± 206.03 ^c^	0.89 ± 0.12 ^a^	0.80 ± 0.22 ^a^	356.16 ± 160.13 ^c^	322.69 ± 163.74 ^c^	0.32 ± 0.45 ^a^
WPI-CS:CO (15:5)	2732.06 ± 534.09 ^a^	0.82 ± 0.01 ^a^	0.41 ± 0.13 ^b^	1129.16 ± 233.24 ^a^	924.43 ± 193.95 ^a^	0.15 ± 0.01 ^a^
WPI-CS:CO (13:7)	1630.14 ± 357.03 ^b^	0.86 ± 0.07 ^a^	0.53 ± 0.23 ^b^	805.40 ± 118.35 ^b^	698.00 ± 145.82 ^b^	0.15 ± 0.07 ^a^
WPI-CS:CO (11:9)	1211.66 ± 212.06 ^b^	0.93 ± 0.21 ^a^	0.52 ± 0.05 ^b^	626.91 ± 114.92 ^b^	587.25 ± 180.61 ^b^	0.22 ± 0.03 ^a^

Note: Data denote mean ± SD. Means in same column with different superscript letters are significantly different at *p* < 0.05.

## Data Availability

The original contributions presented in this study are included in the article; further inquiries can be directed to the corresponding author.
